# CXCR4 antagonism corrects neutrophil abnormalities and reduces pneumonia severity in a pharmacological mouse model of CXCR2 loss-of-function-mediated neutropenia

**DOI:** 10.3389/fimmu.2025.1658987

**Published:** 2025-12-10

**Authors:** Chi Huu Nguyen, Katarina Zmajkovicova, Angelina Sekirnik, Sarah Taplin, Myriam Defontis, Jacob R. Bledsoe, Arthur G. Taveras, Lars Karlsson, Robert Johnson

**Affiliations:** 1 X4 Pharmaceuticals (Austria) GmbH, Vienna, Austria; 2 Research & Development Consultant for X4 Pharmaceuticals, Ljubljana, Slovenia; 3 Integrated Biologix GmbH, Basel, Switzerland; 4 Defontis Veterinary Clinical Pathology, Bully, France; 5 Department of Pathology, Boston Children’s Hospital, Harvard Medical School, Boston, MA, United States; 6 X4 Pharmaceuticals Inc., Boston, MA, United States

**Keywords:** severe congenital neutropenia, neutrophil, CXCR2 loss of function, CXCR4 gain of function, CXCR4 antagonism, preclinical study, infection

## Abstract

**Background:**

The CXCR4 and CXCR2 chemokine receptor axes play critical but opposing roles in regulating neutrophil retention and release from the bone marrow (BM). Gain-of-function (GOF) variants in CXCR4 are associated with WHIM syndrome, characterized by neutropenia, lymphopenia, frequent infections, warts, and myelokathexis. Similarly, loss-of-function (LOF) variants in CXCR2 also result in neutropenia, increased infection susceptibility and myelokathexis. Mavorixafor, an orally bioavailable CXCR4 antagonist, has shown meaningful increases in absolute neutrophil count and reduced infections in WHIM syndrome patients. However, it remains unclear whether CXCR4 antagonism can mitigate the pathogenic characteristics observed in individuals with CXCR2 LOF mutations.

**Methods:**

This study investigated the effects of chronic oral administration of a CXCR4 antagonist on neutrophil abnormalities and infection susceptibility in a CXCR2 LOF mouse model. Mice received the CXCR2 antagonist navarixin orally and then the CXCR4 antagonist compound 1 or vehicle control daily for 7 days. Blood and BM samples were collected for analysis. Treated mice were inoculated with *Streptococcus pneumoniae* to induce pneumonia. Lung tissues were harvested to assess bacterial load and neutrophil counts, and overall survival was monitored.

**Results:**

Pharmacologically induced CXCR2 LOF in mice recapitulated multiple phenotypic features analogous to those observed in patients with CXCR2 LOF, including peripheral blood neutropenia, an elevated myeloid/erythroid ratio (M/E ratio), and neutrophil accumulation with myelokathexis-like (MK-like) morphology in BM, and increased pneumonia susceptibility. Treatment with the CXCR4 antagonist resulted in the correction of these pathologic features, as evidenced by normalization of absolute neutrophil count in peripheral blood, reversal of neutrophil accumulation in BM, normalization of the M/E ratio in BM and reduced the frequency of MK-like neutrophils in BM, and the incidence of myelokathexis. Furthermore, CXCR4 antagonism ameliorated the severity of pneumonia and facilitated the emigration of neutrophils into infected tissues in the CXCR2 LOF mice.

**Conclusions:**

Our findings provide evidence that oral administration of a CXCR4 antagonist can effectively correct blood and BM neutrophil abnormalities and reduce infection susceptibility in a CXCR2 LOF mouse model. These findings suggest potential therapeutic benefits of CXCR4 antagonist therapy in addressing peripheral blood neutropenia and other pathogenic phenotypes in patients with CXCR2 LOF variants.

## 1 Introduction

Severe congenital neutropenia (SCN) is a rare hematological disorder characterized by markedly reduced levels of circulating neutrophils, which significantly increases the susceptibility of affected individuals to recurrent infections (1–3). The pathogenesis of SCN is primarily linked to genetic variants that disrupt the differentiation, production, and egress of neutrophils from the bone marrow (BM) (1–3). Notably, genetic variants in the chemokine receptors C-X-C chemokine receptor 4 (CXCR4) or C-X-C chemokine receptor 2 (CXCR2) have been identified as key contributors to the impaired release of neutrophils from the BM into peripheral blood in a subset of patients with SCN (2–4).

CXCR4 plays a crucial role in retaining neutrophils within BM by interacting with its ligand, CXCL12, which is abundantly produced by osteoblasts and endothelial cells in BM (5, 6). Gain-of-function (GOF) variants in the CXCR4 gene are primarily associated with WHIM syndrome, a condition that falls under the umbrella of SCN (7–10). Patients with WHIM syndrome often experience neutropenia, frequent infections, and the accumulation of neutrophils in BM, with distinctive morphology termed myelokathexis. They may also present with warts, hypogammaglobulinemia and lymphocytopenia (7, 11, 12). In contrast, CXCR2 serves an antagonistic role to CXCR4 (13). Activation of CXCR2 by the CXCL8 subfamily of chemokines, such as CXCL1 and CXCL2, promotes the mobilization of neutrophils from the BM into circulation (14–17). CXCR2 is also crucial for the rapid migration of neutrophils to sites of infection or inflammation, ensuring an effective immune response (18–21). Conversely, patients with loss-of-function (LOF) variants in CXCR2 exhibit many phenotypic traits similar to those seen in individuals with CXCR4 GOF mutations, such as neutropenia, increased susceptibility to infections and myelokathexis (22–25). The precise prevalence or incidence of CXCR2 deficiency linked to neutropenia in the general population is currently unknown. However, it is estimated to be less than 1 in 1,000,000 (https://www.orpha.net/en/disease/detail/420699). To date, nine cases have been documented in the literature through individual case reports and patient registries (22–26).

Given the significant role of CXCR4 GOF variants in the pathogenesis of WHIM syndrome, CXCR4 antagonists have emerged as a promising therapeutic strategy (27–32). The orally bioavailable CXCR4 antagonist mavorixafor has been shown to effect clinically meaningful improvements in peripheral blood neutrophil and lymphocyte counts, and reductions in infection rates among patients aged 12 and older with WHIM syndrome (32, 33), leading to the approval of Xolremdi^®^ by the U.S. Food and Drug Administration. Recent phase 2 clinical proof of concept studies also indicate that mavorixafor can sustain increases in absolute neutrophil counts in individuals suffering from chronic neutropenia, further highlighting its potential therapeutic benefits (34). However, despite these encouraging findings, there remains a gap in understanding whether CXCR4 antagonists can effectively address peripheral blood neutropenia, abnormal BM neutrophil counts, and infection susceptibility specifically in patients harboring CXCR2 LOF variants.

Preclinical studies have suggested that while CXCR4 and CXCR2 receptors and their respective ligands regulate neutrophil release from BM, CXCR4 appears to play a dominant role in this process (13). Additionally, neutrophil mobilization from BM by CXCR2 chemokine CXCL2 is dependent on CXCR4 signaling (35). This observation suggests that inhibiting CXCR4 signaling may effectively correct neutrophil abnormalities in both blood and BM in patients with CXCR2 LOF variants. Therefore, this study aimed to thoroughly investigate the effects of chronic administration of an orally bioavailable CXCR4 antagonist on the pathogenic phenotypes such as abnormalities in blood and BM neutrophil levels, and infection susceptibility, in a pharmacologically induced CXCR2 LOF mouse model.

## 2 Methods

### 2.1 Mouse stain

6-to 8-weeks-old female BALB/c mice, weighing approximately 18–20 g were purchased from Shanghai Lingchang Biotech Co., LTD or Zhejiang Vital River Laboratory Animal Technology Co., Ltd. All *in vivo* mouse experiments were conducted at ChemPartner and WuXi AppTec (Shanghai, China) from November 2023 to April 2025. Studies were performed according to the protocol approved by animal facilities and the Institutional Animal Care and Use Committee (IACUC) of Shanghai ChemPartner and WuXi AppTec following the guidance of the Association for Assessment and Accreditation of Laboratory Animal Care (AAALAC). All procedures of feeding, management, using, and euthanasia for the experimental animals strictly complied with the relevant regulations of AAALAC.

Mice were allowed to acclimate for at least 5 days before the experiment started. Mice were housed at a density of 6 mice per cage in individually ventilated cages within a specific pathogen-free animal facility. All cages, bedding, and water were autoclaved to maintain sterility, with replacements occurring at least once per week. Environmental parameters were strictly controlled, maintaining a temperature of 20–26 °C, relative humidity of 40–70%, and a computer-controlled 12-hour light-dark cycle. Throughout the study, animals had unrestricted access to irradiation-sterilized dry granule food and sterile drinking water. Each cage was labeled with key information, including the number of animals, sex, strain, date of arrival, treatment details, study number, group number, and the start date of the treatment. Individual mice were marked with ear coding for traceability. Littermates were distributed as evenly as possible across experimental groups to reduce any potential confounding effects related to microbiome composition. All mice within the same cage received the same treatment.

### 2.2 CXCR2 and CXCR4 antagonist treatment

Mice were orally gavaged with the CXCR2 antagonist navarixin at 3 mg/kg/day (MedChemExpress) and subsequently with the CXCR4 antagonist compound 1 at 10 mg/kg/day (X4 Pharmaceuticals) daily for 7 days, 2 hours after navarixin dosing. Vehicle only was given as control in all experiments (10% DMSO + 40% PEG300 + 5% Tween80 + 45% saline and 50 mM citrate Buffer, pH4.0). Compound 1 is an orally bioavailable, small molecule CXCR4 antagonist of molecular weight ~400 g/mol. Blood samples were collected from the mice every 7 days, 4 hours post-dosing, for complete blood cell count analysis using a Sysmex XN-350 hematology analyzer (Sysmex). Peripheral blood was obtained via submandibular puncture. BM samples were collected 4 hours after the final Compound 1 dose on day 7 for numeration and flow cytometry analysis. Euthanasia of the mice was conducted using a gradual increase in CO_2_, with a flow rate set to approximately 50% of the chamber volume per minute.

For BM single-cell suspensions, femora and tibiae were flushed with DPBS (Corning). Cell collection was carried out in DPBS supplemented with 5% fetal bovine serum (ExCell Bio) and filtered through a 70-μm nylon strainer to eliminate debris and fat. Red blood cell lysis was performed using ACK Lysis Buffer (Thermo Fisher). The total number of BM suspension cells was quantified using a Countstar^®^ Rigel S2 cell counter (ALIT).

For lung single-cell suspensions, lung tissue was rinsed in ice-cold DPBS and chopped with scissors. The minced tissue was then incubated with a dissociation mix containing 20 μg/mL DNase I (Tiangen) and 1 mg/mL Collagenase (Sigma), diluted in DMEM (Gibco), at 37 °C for 30 minutes, with shaking every 10 minutes. Following enzymatic digestion, the tissue was further dissociated using a gentleMACS Octo Dissociator (Miltenyi Biotec). The resulting cell suspension was filtered through a 70 μm strainer to obtain a single-cell suspension. Red blood cells were lysed using ACK Lysis Buffer, and the suspended cells were washed twice with cold DPBS. Finally, the cells were counted using Countstar^®^ Rigel S2, and appropriate aliquots were collected for flow cytometry analysis.

### 2.3 Flow cytometry

Single-cell suspensions were initially stained with Live/Dead (R3) dye to differentiate between live and dead cells, followed by incubation with an Fc blocker (BioLegend) for 5 minutes at 4°C in the dark. Subsequently, cells were stained with specific antibodies in a cell staining buffer (BioLegend or eBioscience) for 30 min at 4°C in the dark. The list of antibodies used in the study is shown in **Supplementary Table S1**. In some experiments, stained samples were fixed with a Fix buffer (BD Biosciences) before analysis. Analyses were performed using either the Attune NxT V6 (Thermo Fisher) or the FACSCanto II Plus (BD Biosciences), with data analyzed using FlowJo software (TreeStar).

### 2.4 Bone marrow and blood smear analysis

Peripheral blood and BM aspirate were collected 4h after last dosing on day 7. Blood and BM smears were stained with May-Grunwald Giemsa (Sigma MG500, Sigma-Aldrich), and evaluated under light microscopy using a BX 43 Olympus microscope with images captured using a x100 objective. Blood smears were assessed at low magnification (x10) for a qualitative analysis of total neutrophil counts. Subsequently, high magnification (x50 and x100, oil immersion) was used to evaluate the monolayer area of the blood smear and the morphology of the neutrophils. For BM smears, two BM smears per animal were examined microscopically. The smears were evaluated at low magnification (x10) for their overall quality (cellularity, smearing artifacts, cell lysis, blood contamination, etc.). Then, for smears of adequate quality, approximately 15 to 20 high-power fields were evaluated at high magnification (x50 and x100, oil immersion). The hematopoietic cells were evaluated for their maturation sequences and morphology, the number of myeloid and erythroid cells was counted and a myeloid to erythroid ratio was calculated.

### 2.5 Infection of mice with *Streptococcus pneumoniae*

*Streptococcus pneumoniae 6301* (ATCC) was grown overnight at 37°C and 5% CO_2_ in Tryptic Soy Agar (TSA; Hopebio) supplemented with 5% sterile defibrillated sheep blood (Guanzhou Ruite Biotechnology Co., Ltd.). The bacteria were then transferred to nutrient broth (NB) liquid medium supplemented with 10% horse serum (Guanzhou Ruite Biotechnology Co., Ltd) and incubated overnight under the same conditions. Following this, the bacteria were subcultured for approximately 4 hours in NB liquid medium to reach the mid-logarithmic phase for inoculum preparation.

Mice were treated with CXCR2 and CXCR4 antagonists as outlined in Section 2.2, from day 0 through day 14. On day 1 of the treatment protocol, immediately after administration of the CXCR2 and CXCR4 antagonists, the mice were anesthetized using isoflurane and subsequently inoculated intratracheally with *Streptococcus pneumoniae* at a dose of 3 × 10^4^ CFU per mouse to establish a lung infection model. Following the inoculation, lung tissue samples were collected at 24 and 72 hours post-infection to assess bacterial counts and the recruitment of neutrophils, respectively. In some cohorts, mice were daily monitored until day 14 post-infection to assess overall survival.

### 2.6 Determination of bacterial loads in lung tissue homogenate

Bacterial loads in the lungs of mice infected with *Streptococcus pneumoniae* were assessed across various experimental groups by analyzing lung tissue homogenates. Mice were euthanized using CO_2_, after which their lungs were excised, weighed, and homogenized in DPBS. To quantify the bacterial burden, ten-fold serial dilutions of the lung tissue homogenates were prepared and plated on TSA supplemented with 5% sheep blood. The plates were then incubated at 35 ± 2°C in a CO_2_ incubator overnight to facilitate bacterial growth. The actual bacterial load of each organ was calculated according to the corresponding dilution titers.

### 2.7 Statistical analysis

The data were presented as mean ± standard error of the mean (SEM) and the number of mice per experiments was indicated in the figure legends. All statistical analyses were conducted using Prism software (GraphPad Software, Boston, MA, USA). The significance of differences between two independent groups was calculated using Fisher’s exact test or Chi-square test. The significance of differences between multiple groups was determined by two-way ANOVA followed by Bonferroni *post hoc* test. The log-rank test was used to evaluate survival differences between groups of mice. P values <0.05 were considered statistically significant.

## 3 Results

### 3.1 CXCR4 antagonism corrects peripheral blood neutropenia in a pharmacological mouse model of CXCR2 LOF

**Figure 1:**
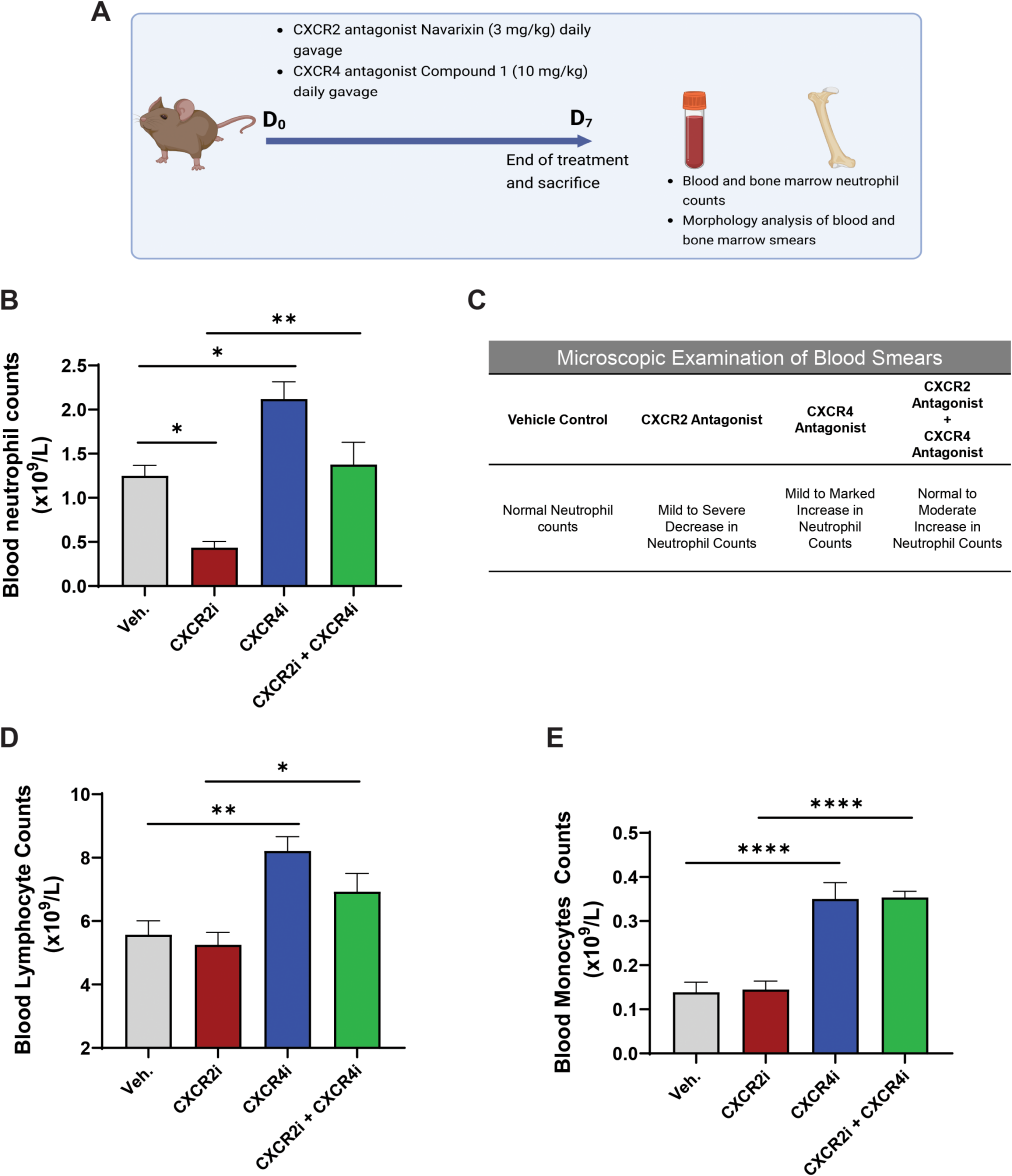
CXCR4 antagonism corrects peripheral blood neutropenia in a pharmacological mouse model of CXCR2 LOF. **(A)** Experimental design: BALB/c mice were treated with the CXCR2 antagonist navarixin to establish a pharmacological CXCR2 LOF model, followed by treatment with the CXCR4 antagonist compound 1. Blood and BM were collected for analysis. Figure was created using the Biorender software (Biorender.com, agreement number: E61C43FA-0001). **(B)** Absolute neutrophils counts were determined in the blood of CXCR2 and CXCR4 antagonist-treated mice 4 hours after the last dose on day 7. **(C)** Summary of microscopic examination of blood smears collected from CXCR2 and CXCR4 antagonist-treated mice 4 hours after the last dose on day 7. Absolute lymphocyte (**D**), and monocyte counts **(E)** were determined in the blood of CXCR2 and CXCR4 antagonist-treated mice 4 hours after the last dose on day 7. Data are represented as mean + SEM with 6 mice per group. Statistics were calculated using the two-way ANOVA followed by Bonferroni *post hoc* test. *p < 0.05, **p < 0.01, ****p < 0.00001. CXCR4, C-X-C chemokine receptor 4; CXCR2, C-X-C chemokine receptor 2; SEM, standard error of the mean; Veh., vehicle; CXCR4i, CXCR4 antagonist; CXCR2i, CXCR2 antagonist.

To investigate whether CXCR4 antagonism can correct peripheral blood neutropenia, a common clinical phenotype observed in patients with CXCR2 LOF variants (22–25), we initially considered using *Cxcr2* knockout (*Cxcr2^-/-^*) mice. However, *Cxcr2^-/-^* mice do not fully replicate the pathogenic phenotypes seen in patients with CXCR2 LOF, as they exhibit peripheral blood neutrophilia, extramedullary hematopoiesis, altered distribution and activation state of retinal glia, and deficits in electrical retinal responses and visual acuity (26, 36, 37). To address this limitation, we employed a pharmacological approach to impair CXCR2 signaling and explore the impact of CXCR4 antagonism on pathogenic phenotypes. To this end, mice were treated with the CXCR2 antagonist navarixin (3 mg/kg) and subsequently with the orally bioavailable CXCR4 antagonist compound 1 (10 mg/kg) by daily gavage for 7 days. Blood cell counts were measured 4 hours after the last dose (**Figure 1A**). Compound 1 exhibited a robust and sustained CXCR4 antagonism, as evidence by its potent inhibition of CXCL12 ligand binding (IC_50_ = 0.7 nM), CXCL12-mediated calcium flux (IC_50_ = 3.6 nM), and CXCL12-mediated cell migration (IC_50_ = 7.0 nM), along with a receptor occupancy half-life of 36 minutes (**Supplementary Table S2**). A PathHunter^®^ β-Arrestin screening assay assessing β-arrestin recruitment to chemokine receptors suggested that compound 1 exhibited high selectivity for CXCR4 in comparison to other chemokine receptors (**Supplementary Table S3**). Pharmacokinetic parameters following a single administration of compound 1 are summarized in **Supplementary Table S4**.

Treatment with the CXCR2 antagonist resulted in profound peripheral blood neutropenia, evidenced by a ~65% reduction in circulating neutrophil levels compared to control mice (**Figure 1B**). The CXCR2 antagonist did not impact circulating lymphocyte, monocyte, red blood cell, or platelet counts (**Figures 1D, E**, **Supplementary Figures 1A, B**). This observation aligns with the hematological phenotypes observed in patients with CXCR2 LOF variants (22–25), suggesting that pharmacologically induced CXCR2 LOF replicates the neutropenia phenotype seen in patients. Hence, mice treated with the CXCR2 antagonist are referred to as CXCR2 LOF mice hereafter.

Importantly, CXCR4 antagonist treatment led to a significant increase in absolute neutrophil count and corrected circulating neutropenia in CXCR2 LOF mice (**Figure 1B**). Blood smear analysis corroborated these results, confirming that the CXCR2 LOF mice exhibited circulating neutropenia, which was reversed by CXCR4 antagonism (**Figure 1C**). Consistent with previous preclinical models (38–41), treatment with the CXCR4 antagonists also led to increases in the absolute numbers of neutrophils, lymphocytes, and monocytes in control mice (**Figures 1B, D, E**). While CXCR4 antagonism corrected peripheral blood neutropenia in CXCR2 LOF mice (**Figures 1B-E**), it did not appear to affect the count of red blood cells or platelets in both CXCR2 LOF and control mice (**Supplementary Figures 1A, B**), consistent with a previous report (38).

Altogether, these results indicate that CXCR4 antagonism corrected peripheral blood neutropenia in CXCR2 LOF mice.

### 3.2 CXCR4 antagonism normalizes neutrophil accumulation in bone marrow and reduced splenic neutrophil count in a pharmacological mouse model of CXCR2 LOF

Impaired CXCR2 signaling has been demonstrated to lead to increased retention of neutrophils in BM and an elevated myeloid-to-erythroid (M/E) ratio, which are characteristic features observed in patients with CXCR2 LOF variants (22, 24). Given these significant associations, we next investigated whether our pharmacologically induced CXCR2 LOF mouse model exhibited similar BM phenotypes and evaluated the effects of CXCR4 antagonism on these abnormalities.

**Figure 2:**
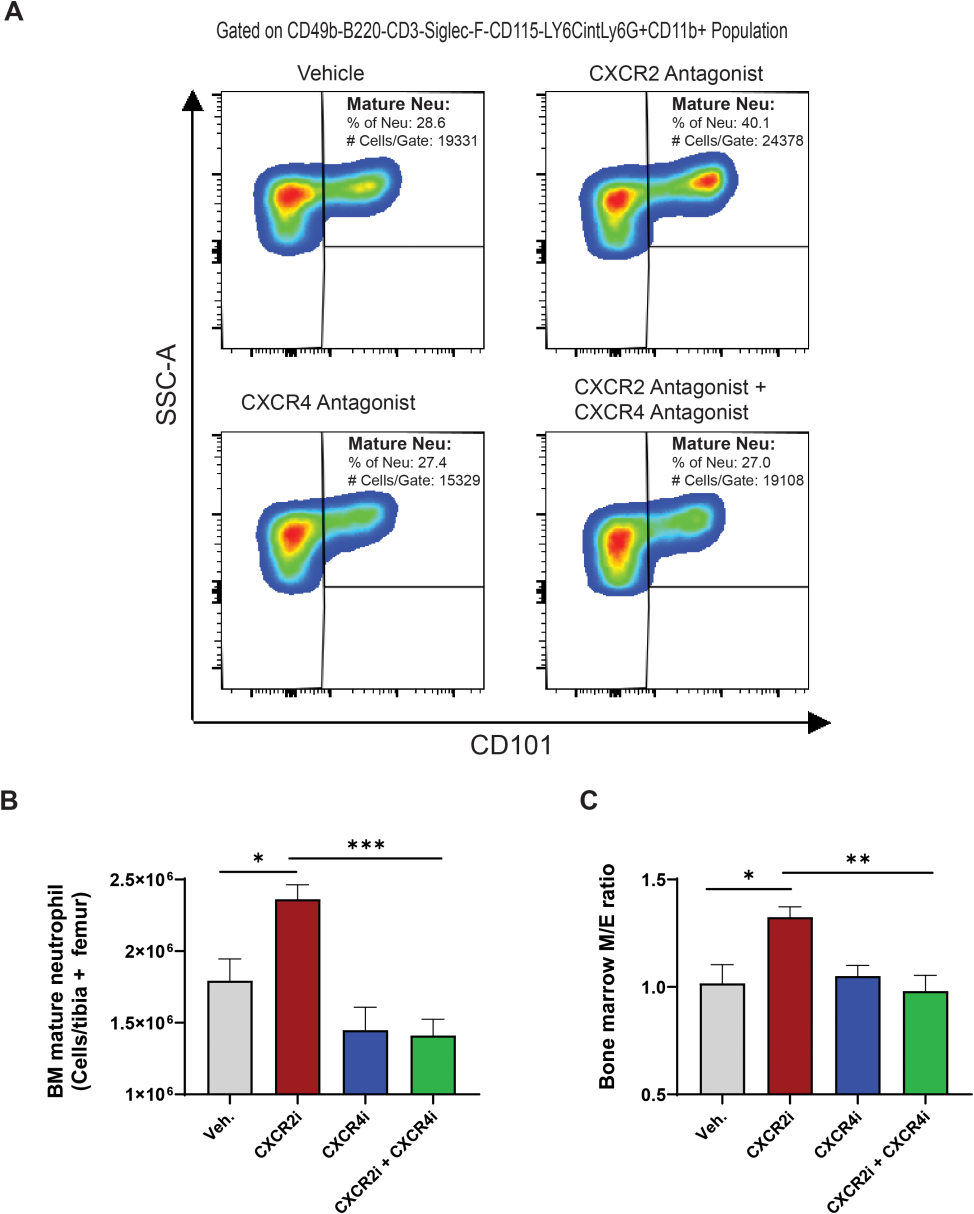
CXCR4 antagonism normalizes neutrophil accumulation in BM in a pharmacological mouse model of CXCR2 LOF. **(A)** Representative flow cytrometric analysis of mature neutrophil in BM from mice treated with CXCR2 and CXCR4 antagonists. Samples were collected 4 hours after the final dose on day 7. **(B)** Absolute counts of mature neutrophils and **(C)** M/E ratio in BM of CXCR2 and CXCR4 antagonist-treated mice were determined 4 hours after the last dose on day 7. BM mature neutrophils were defined as CD49b^-^B220^-^CD3^-^Siglec-F^-^CD115^-^LY6C^int^Ly6G^+^CD11b^+^CD101^+^. Data are represented as mean + SEM with 6 mice per group. Statistics were calculated using the two-way ANOVA followed by Bonferroni *post hoc* test. *p < 0.05, **p < 0.01, ***p < 0.0001. CXCR4, C-X-C chemokine receptor 4; CXCR2, C-X-C chemokine receptor 2; SEM, standard error of the mean; Veh., vehicle; CXCR4i, CXCR4 antagonist; CXCR2i, CXCR2 antagonist.

Our CXCR2 LOF mice exhibited a markedly higher count of mature neutrophils in BM and an increased M/E ratio compared to control mice (**Figures 2A-C**). Furthermore, we observed no significant differences in the counts of immature neutrophils, as well as hematopoietic stem and progenitor cell subset counts between CXCR2 LOF and control mice (**Supplementary Figures 2A-E**). This finding suggests that the observed accumulation of mature neutrophils is not due to enhanced expansion but is likely a consequence of their impaired egress from BM resulting from CXCR2 blockade. Interestingly, treatment with the CXCR4 antagonist significantly normalized the accumulation of mature neutrophils in BM and led to a correction of the M/E ratio in CXCR2 LOF mice (**Figures 2A-C**). Consistent with previous findings (38, 41, 42), treatment with the CXCR4 antagonist led to a decrease in BM mature neutrophil counts (**Figures 2A-B**) while simultaneously increasing their numbers in peripheral blood (**Figure 1B**) in control mice. This supports the notion that BM serves as an important reservoir for neutrophils mobilized by CXCR4 antagonism.

In addition to BM, the spleen acts as a potential reservoir for neutrophils and is classically considered a key site for their clearance (43, 44). Immunohistochemistry staining of spleen sections indicated that CXCR2 LOF mice exhibited significantly reduced splenic neutrophil counts compared to control mice. Furthermore, treatment with a CXCR4 antagonist led to an increase and normalization of splenic neutrophil counts in CXCR2 LOF mice (**Supplementary Figures S3A, B**). Notably, CXCR4 antagonism also resulted in an increase in splenic neutrophil counts in control mice (**Supplementary Figures S3A, B**), aligning with previous findings (45, 46).

In summary, our findings indicate that CXCR4 antagonism effectively reverses the accumulation of mature neutrophils in BM and normalizes the M/E ratio as well as splenic neutrophil counts in CXCR2 LOF mice.

### 3.3 CXCR4 antagonism reduces incidence of myelokathexis phenotype in a pharmacological mouse model of CXCR2 LOF

**Figure 3:**
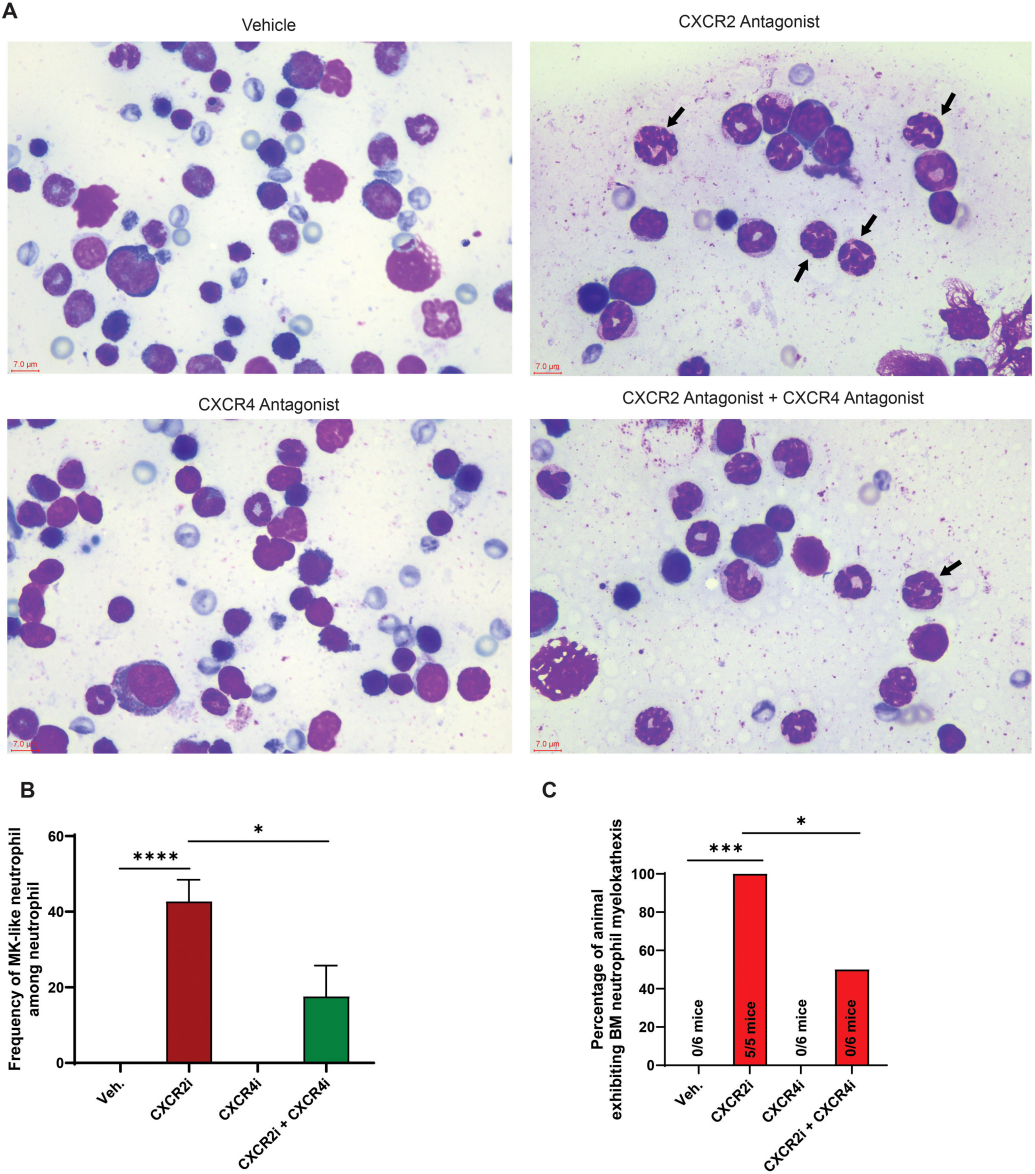
CXCR4 antagonism reduces incidence of myelokethaxis phenotype in a pharmacological mouse model of CXCR2 LOF. **(A)** BM smears from CXCR2 and CXCR4 antagonist-treated mice were collected 4 hours after the last dose on day 7 and stained with May-Grunwald Giemsa. Arrows indicate neutrophils exhibiting myelokathexis-like morphologic features, characterized by nuclear hypersegmentation, an increased number of discernible nuclear lobes sometimes separated by thin chromatin strands. Scale bar: 7 µm. **(B)** The frequency of MK-like neutrophil among neutrophil in BM smears of CXCR2 and CXCR4 antagonist-treated mice. Data are represented as mean + SEM with 5–6 mice per group. Statistics were calculated using the two-way ANOVA followed by Bonferroni *post hoc* test. *p < 0.05, ****p<0.0001. **(C)** Percentage of mice within the cohort displaying neutrophil myelokethaxis in BM. The number shown on each bar represents the count of mice exhibiting the MK-like phenotype. Statistics were calculated using Chi-square test. *p < 0.050, ***p<0.001. CXCR4, C-X-C chemokine receptor 4; CXCR2, C-X-C chemokine receptor 2; Veh., vehicle; CXCR4i, CXCR4 antagonist; CXCR2i, CXCR2 antagonist.

Myelokathexis is characterized by the abnormal retention of neutrophils in BM with distinct neutrophil morphology, including condensed chromatin with long, thin, often redundant chromatin strands separating nuclear lobes (7, 12, 47, 48). This condition is a diagnostic feature in BM and blood of patients with CXCR4 GOF variants (7, 12, 47–49) and has also been observed in a subset of patients exhibiting LOF variants in CXCR2 (23–25). Consistent with observations in patients with CXCR4 GOF or CXCR2 LOF, myelokathexis-like (MK-like) neutrophils, characterized by nuclear hypersegmentation, an increased number of discernible nuclear lobes, and thinner intranuclear chromatin strands, were identified at a significantly elevated frequency in the BM of pharmacologically induced CXCR2 LOF mice, in contrast to control mice (**Figures 3A, B**). Qualitative assessment estimated frequency of MK-like neutrophil among neutrophil in BM at approximately 43% in CXCR2 LOF mice, whereas no such cells were detected in control animals (**Figure 3B**). Notably, MK-like neutrophils were present in BM of all CXCR2 LOF mice (N = 5/5, 100%) (**Figure 3C**). Administration of the CXCR4 antagonist over seven days significantly diminished both the frequency of MK neutrophils in the BM (to approximately 17%), and the overall number of mice exhibiting neutrophil myelokathexis (N = 3/6, 50%) (**Figures 3B, C**). MK-like neutrophils were also detected in the peripheral blood of pharmacologically induced CXCR2 LOF mice, but not in control mice (**Supplementary Figures 3A, B**). These MK-like neutrophils constituted approximately 7% of the total blood neutrophils in CXCR2 LOF mice (**Supplementary Figures 3A, B**). Additionally, the morphology and frequency of MK-like neutrophils in the blood of CXCR2 LOF mice remained unchanged from baseline levels following one week of treatment with the CXCR4 antagonist, consistent with our previous findings in CXCR4 GOF mouse model (49, 50).

Taken together, our data indicate that CXCR4 antagonism reduces the frequency of MK neutrophils in the BM and the incidence of myelokathexis phenotype in a pharmacological mouse model of CXCR2 LOF.

### 3.4 CXCR4 antagonism reduces pneumonia severity in a pharmacological mouse model of CXCR2 LOF

Patients with CXCR2 LOF variants often exhibit increased susceptibility to upper respiratory infections (22, 24). Previous preclinical studies have demonstrated that *Cxcr2*^-/-^ mice, as well as mice subjected to pharmacological blockade of the CXCR2 receptor using CXCR2 antagonists or those deficient in the CXCR2 chemokine CXCL1, show heightened vulnerability to challenges with *Streptococcus pneumoniae* (20, 51). Therefore, we finally sought to investigate whether our pharmacologically induced CXCR2 LOF mice also display increased susceptibility to *Streptococcus pneumoniae* infection and whether CXCR4 antagonism could mitigate this susceptibility.

**Figure 4:**
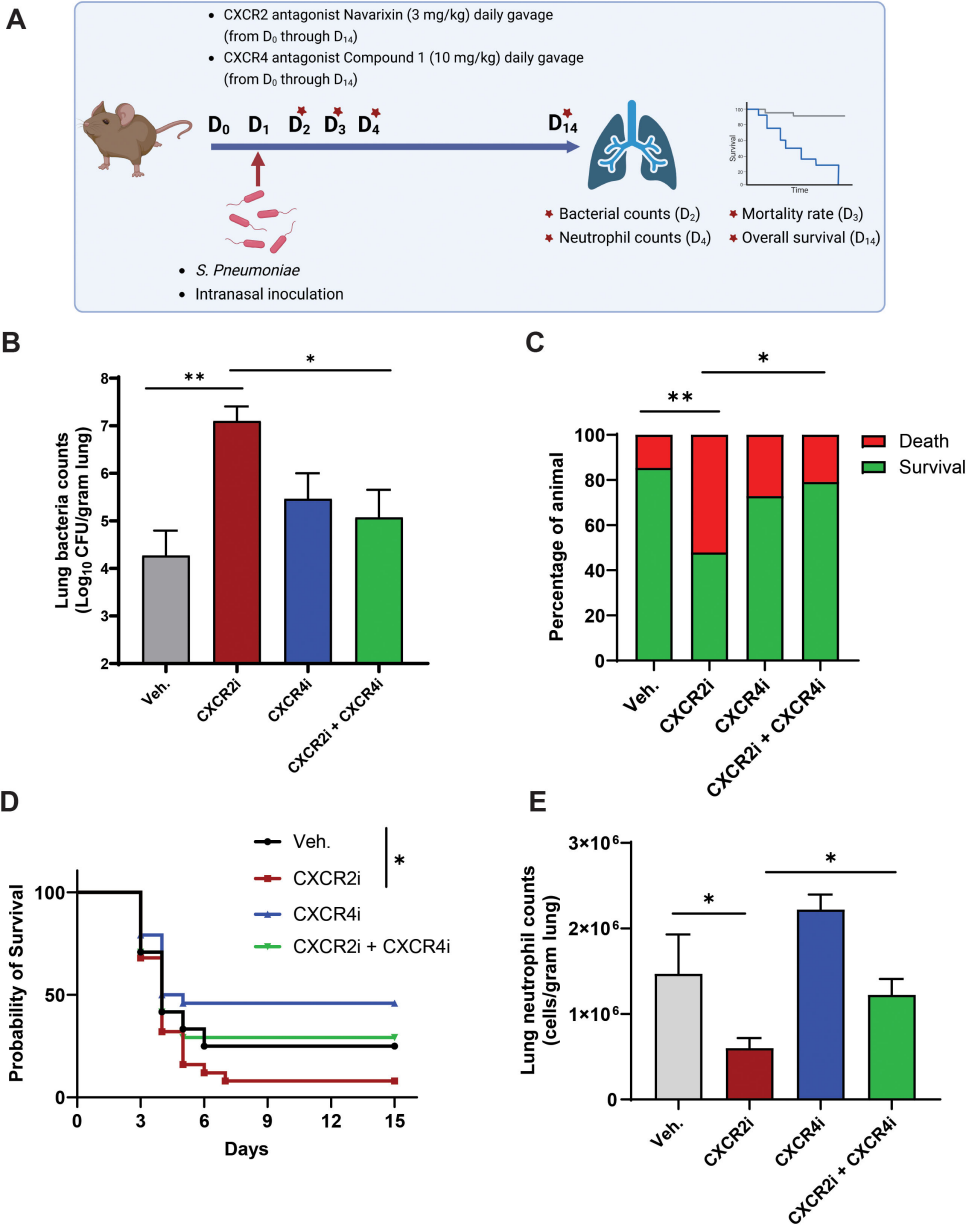
CXCR4 antagonism reduces pneumonia severity in a pharmacological mouse model of CXCR2 LOF. **(A)** Experimental design for *Streptococcus Pneumonia* infection model. Mice were treated with CXCR2 and CXCR4 antagonist and then inoculated with *Streptococcus pneumonia* before analysis of bacterial burden, neutrophil count and mortality. Figure was created using the BioRender software (Biorender.com, agreement number: E61C43FA-0001). **(B)** The bacterial burden in lung tissue homogenates was quantitated at 24 hours post-infection. **(C)** The mortality rate was determined at 48 hours post-infection. Data were from two independent experiments with 20 mice/group. Statistics were calculated using Fisher’s exact test. *p < 0.05, **p < 0.01. **(D)** Survival was monitored up to 15 days. A Kaplan-Meier plot is used to show survival of mice from each group. Summary of two independent experiment with N = 24 mice/group; *p < 0.05; log-rank test. **(E)** The total neutrophil counts in lung tissue homogenates were quantified at 72 hours post infection. **(B, E)** Data (mean + SEM) were from two independent experiments with 9–12 mice per group. Statistics were calculated using by two-way ANOVA followed by Bonferroni *post hoc* test. *p < 0.05, **p < 0.01. The difference between Vehicle and CXCR4 antagonist alone in **Figures 4B-D** is insignificant. CXCR4, C-X-C chemokine receptor 4; CXCR2, C-X-C chemokine receptor 2; SEM, standard error of the mean; Veh., vehicle; CXCR4i, CXCR4 antagonist; CXCR2i, CXCR2 antagonist.

To this end, mice were treated with the CXCR2 antagonist navarixin (3 mg/kg) and the CXCR4 antagonist compound 1 (10 mg/kg) by daily oral gavage from day 0 through day 14. On day 1 of treatment protocol, immediately after administering the CXCR2 and CXCR4 antagonists, mice were subsequently challenged with *Streptococcus pneumoniae*. Bacterial load and total neutrophil counts were analyzed, and mortality as well as overall survival were monitored (**Figure 4A**). In line with previous reports (20, 21, 51), pharmacologically induced CXCR2 LOF mice exhibited a significantly increased bacterial load in lung tissue homogenates compared to control mice at day 1 post-pneumococcal challenge (**Figure 4B**). Additionally, CXCR2 LOF mice displayed progressive mortality, with a 46% mortality rate by day 2 post-infection, compared to 23% in control mice. Notably, treatment with the CXCR4 antagonist effectively reduced both bacterial load in lung tissues and the mortality rate in CXCR2 LOF mice, while having no significant effect on these parameters in control mice (**Figures 4B, C**). When mice were monitored up to 14 days post-infection, CXCR2 LOF mice also exhibited significantly reduced overall survival compared to control mice, and this reduction appeared to improve with CXCR4 antagonist treatment (**Figure 4D**). Moreover, previous studies have indicated that the increased susceptibility to infection observed in *Cxcr2*^-/-^ and *Cxcl1*^-/-^ mice is linked to impaired neutrophil recruitment into infected tissues (20, 21, 51, 52). Our findings corroborate this, as total neutrophil counts in lung tissue homogenates were diminished in CXCR2 LOF mice compared to controls. Treatment with the CXCR4 antagonist restored neutrophil counts in lung tissues (**Figure 4E**), suggesting that CXCR4 antagonism may normalize neutrophil infiltration into infected tissues, thereby facilitating bacterial clearance.

In conclusion, our data indicate that CXCR4 antagonism reduces pneumonia severity in a pharmacological mouse model of CXCR2 LOF, and this effect is likely associated with the normalization of neutrophil numbers in infected tissues.

## 4 Discussion

In this study, we investigated the impact of a CXCR4 antagonist on the pathogenic phenotypes seen in patients with chronic neutropenia, including abnormalities in blood and BM neutrophil levels, as well as infection susceptibility, in a pharmacologically induced CXCR2 LOF mouse model. Our findings demonstrate that CXCR4 antagonism effectively corrected peripheral blood neutropenia, restored reduced splenic neutrophil counts, normalized mature neutrophil accumulation in BM, and normalized the M/E ratio and MK-like phenotype in BM of CXCR2 LOF mice. Additionally, CXCR4 antagonism mitigated the severity of pneumonia in CXCR2 LOF mice and facilitated neutrophil emigration into bacterially infected tissues. These findings suggest potential therapeutic benefits of CXCR4 antagonist therapy in reversing peripheral blood neutropenia and other pathogenic phenotypes associated with CXCR2 LOF variants in patients.

To investigate the relationship between CXCR2 LOF variants and pathogenic features, genetic inactivation of CXCR2 in mice would normally be the preferred model. However, the complete genetic ablation of CXCR2 in mice does not replicate the picture seen in patients, resulting in phenotypic discrepancies such as peripheral blood neutrophilia, extramedullary hematopoiesis and altered retinal glial cell distribution (26, 36, 37). These unexpected phenotypes underscore the compensatory mechanisms that may arise from gene deletion, limiting the utility of this model for studying patient phenotypes. In contrast*, Cxcr2^-/-^* mixed BM chimeras exhibit patient-like features, including circulating neutropenia and BM myelokathexis (13), but present challenges like immune interactions, donor cell variability, and complications from conditioning regimens and immunosuppression (53, 54). Alternatively, a pharmacological approach using small molecule inhibitors may offer a more precise and reversible method to study CXCR2-dependent neutrophil trafficking (55–57). In our study, pharmacologically induced CXCR2 LOF in mice displayed pathogenic phenotypes similar to those described in patients with germline CXCR2 LOF variants, such as peripheral blood neutropenia, accumulation of mature neutrophils in BM, increased M/E ratio, neutrophils exhibiting myelokathexis-like morphology, and heightened susceptibility to infections. Previous research has demonstrated that genetic inactivation of *Cxcr2* in mice activates G-CSF production, thereby promoting BM granulopoiesis and increased BM neutrophil counts (58). The observed abnormalities in BM neutrophil counts and M/E ratio in the CXCR2 LOF mouse model are likely due to impaired neutrophil release from the BM rather than enhanced granulopoiesis, as pharmacological inhibition of CXCR2 for a duration of 7 days did not affect BM hematopoietic stem and progenitor cell subsets or immature neutrophil counts. Future studies are needed to determine whether pharmacologically induced CXCR2 LOF would also impact G-CSF production and, consequently, BM and blood neutrophil counts. In addition to the abnormalities in BM and blood neutrophil counts, we also observed a significant reduction in total neutrophil counts in the spleen of pharmacologically induced CXCR2 LOF mice compared to control mice. This reduction may result from a compromised egress of neutrophils from the BM to the bloodstream, and subsequently to the spleen. Interestingly, complete *Cxcr2^-/-^* mice displayed increased splenic neutrophil counts but a decreased percentage of mature splenic neutrophils (26). Conversely, the splenic neutrophil reservoir remained unchanged in *Cxcr2^-/-^* mixed BM chimeric models (13), suggesting that the impact of *Cxcr2* inactivation on splenic neutrophil counts may vary depending on the specific model used. Nevertheless, the observed phenotypes suggest that pharmacologically induced CXCR2 LOF serves as an alternative tool to explore the relationship between CXCR2 LOF variants and pathogenic features.

Neutrophil release from BM to peripheral blood is tightly regulated through the interplay of CXCR4 and CXCR2 signaling (13, 14, 35). Disruptions in either pathway can lead to increased neutrophil retention in the BM and reduced peripheral blood neutrophil levels, a common feature in patients with CXCR4 GOF and CXCR2 LOF variants (7, 12, 22–24). Preclinical studies have shown that CXCR2 signaling is not essential for neutrophil mobilization in the absence of CXCR4, and that neutrophil mobilization via the CXCR2 chemokine CXCL1 is significantly enhanced by transient CXCR4 inhibition, highlighting CXCR4’s dominant role in neutrophil trafficking from the BM (13, 35). Our results further support this finding, demonstrating that chronic treatment with a CXCR4 antagonist increased blood neutrophil counts and corrected peripheral blood neutropenia in CXCR2 LOF mice. Additionally, our data indicated that the CXCR4 antagonist effectively reversed neutrophil accumulation in the BM and normalized the M/E ratio in these mice. These results suggest that selectively blocking the CXCR4 receptor disrupts the retention signals that normally sequester neutrophils within the BM of CXCR2 LOF mice, thereby facilitating their release into circulation. Our observation that CXCR4 treatment reduced BM mature neutrophil counts while increasing their number in blood of both control and CXCR2 LOF mice suggests that the BM is a key source of CXCR4 antagonist-mobilized neutrophils, corroborating previous findings (14, 41, 46). Additionally, continuous dosing with an oral CXCR4 antagonist did not affect the counts of hematopoietic stem and progenitor cells or immature neutrophils in the BM of either control or CXCR2 LOF mice. This indicates that CXCR4 antagonism effectively mobilizes neutrophils into peripheral blood without disrupting BM reserve. In addition to correcting BM and neutrophil count abnormalities, CXCR4 antagonist treatment also restored splenic neutrophil numbers. This may reflect the ability of the CXCR4 antagonist to facilitate the redistribution of neutrophils from primary immune organs to secondary immune sites, as previously reported (41, 45, 46).

Interestingly, a previous study demonstrated that transient treatment with plerixafor (AMD3100), an injectable CXCR4 antagonist, failed to mobilize *Cxcr2^-/-^* neutrophils in a *Cxcr2^-/-^* mixed BM chimeric mouse model (13). This finding contrasts with our data using compound 1, an orally bioavailable CXCR4 antagonist, in a pharmacologically induced CXCR2 LOF mouse model. These observations suggest that the efficacy of CXCR4 antagonists in mobilizing neutrophils and correcting peripheral blood neutropenia may be compound-specific and influenced by several factors such as pharmacokinetic properties, tissue distribution, residence time on CXCR4 receptor, and the duration of antagonist treatment. Supporting this speculation, compound 1, administered chronically over a 7-day period in our study, exhibited an enhanced tissue distribution (V_d_ = 39,200 ml/kg for compound 1 and 477–521 ml/kg for plerixafor), along with a greater half-life compared to plerixafor (T_1/2_ = 6.1 hours for compound 1 versus 0.9-1.16 hours for plerixafor) (**Supplementary Table S4**). Furthermore, compound 1 also displayed a prolonged receptor occupancy, with durations of 36 minutes for compound 1 (**Supplementary Table S2**) and 26 minutes for plerixafor (38). These characteristics may facilitate a more robust and sustained mobilization of neutrophils, effectively correcting circulating neutropenia. However, further studies are necessary to explore this point in more detail.

G-CSF was approved ~30 years ago to treat multiple forms of neutropenia including SCN by elevating circulating absolute neutrophil counts and lowering rates of infection (4, 59). Previous studies indicate that G-CSF-mediated neutrophil mobilization from BM into peripheral blood is dependent on CXCR2 signaling as G-CSF was ineffective in mobilizing neutrophils in *Cxcr2^-/-^* mice (60). Additionally, the ability of G-CSF to mobilize neutrophils and correct blood neutropenia in a *Cxcr2 ^-/-^* mixed BM chimeras mouse model was significantly compromised (13). A similar trend has been observed in healthy volunteers treated with CXCR2 antagonist, albeit to a lesser extent, likely due to the specific CXCR2 antagonist’s affinity and treatment duration (61). These observations suggest that G-CSF may also be less effective in our CXCR2 LOF model, underscoring the need for alternative therapeutic strategies for patients with CXCR2 deficiency. Notably, the correction of peripheral neutropenia observed in our CXCR2 LOF mice following CXCR4 antagonist treatment suggests a promising alternative for patients with CXCR2 LOF who do not adequately respond to G-CSF. This approach may also facilitate a reduction in the frequency and dosage of G-CSF administration, thereby improving patient quality of life and minimizing the associated adverse effects. Future studies will be required to address whether CXCR4 antagonists can correct peripheral blood neutropenia in a *Cxcr2^-/-^* mixed BM chimeras and patients with CXCR2 LOF. Encouragingly, a recent phase 2 proof-of-concept clinical study demonstrated that CXCR4 antagonist, mavorixafor, can elevate neutrophil counts in patients with chronic neutropenia, allowing for reduced G-CSF dosing while maintaining absolute neutrophil counts at normal levels (34). Furthermore, a global, double-blind, placebo-controlled phase 3 clinical trial (NCT06056297) is currently underway, aiming to evaluate the efficacy and safety of mavorixafor in patients with chronic neutropenia, including those with CXCR2 deficiency, both with and without background G-CSF therapy.

Myelokathexis is characterized by the abnormal accumulation of neutrophils in BM, where these neutrophils exhibit a distinct morphology (7, 12, 47). These clinical features are commonly observed in patients with GOF variants of CXCR4 (7, 12, 47–49), and in some patients with LOF variants in CXCR2 (23–25). A MK-like phenotype has been previously reported in BM of *Cxcr2^-/-^* mixed BM chimeras (13). Our pharmacologically induced CXCR2 LOF mouse model showed a high frequency of MK-like neutrophils in the BM, along with a notable increased incidence of mice exhibiting a myelokathexis phenotype. Interestingly, MK-like neutrophils were also detected in the peripheral blood of CXCR2 LOF mice, mirroring observations in a mouse model with CXCR4 GOF (49, 50). Furthermore, our data indicate that treatment with an oral CXCR4 antagonist appeared to reduce the incidence of myelokathexis phenotype and the frequency of MK-like neutrophils in the BM of CXCR2 LOF mice. While chronic treatment with the CXCR4 antagonist appeared to decrease the frequency of MK-like neutrophils in the BM of CXCR2 LOF mice, their frequency in the blood remained unchanged at the end of 7-days of treatment with a CXCR4 antagonist, aligning with prior observations in a mouse model with CXCR4 GOF as well as in patients with CXCR4 GOF variants (47, 49, 50). This suggests that once released from the BM by the CXCR4 antagonist, MK-like neutrophils may redistribute to organs other than peripheral blood, or that newly released MK-like neutrophils have a relatively short half-life, making them difficult to detect in the peripheral blood. An alternative speculation is that CXCR4 antagonists preferentially mobilize morphologically normal neutrophils over those exhibiting myelokathectic features. Nevertheless, further studies are needed to address these questions comprehensively.

Previous preclinical studies have shown that deficiency in *Cxcr2* or its chemokine ligand *Cxcl1* or pharmacological inhibition of these molecules resulted in impaired neutrophil recruitment into infected tissues (20, 21, 51, 52). This impairment increased infection susceptibility to various pathogens, such as *Streptococcus pneumoniae, Klebsiella pneumoniae, Escherichia coli, Staphylococcus aureus*, and *Toxoplasma gondii* (20, 21, 51, 52, 62). Consistent with previous findings, our study revealed that pharmacologically induced CXCR2 LOF with navarixin exhibited increased susceptibility to *Streptococcus pneumoniae* infection, evidenced by increased bacterial load in the lungs, elevated mortality, and decreased overall survival. This increased susceptibility to pneumonia appears to be associated with impaired neutrophil recruitment to the lungs rather than a defect in neutrophil function itself. Supporting this notion, chronic treatment with CXCR2 antagonists had no adverse effect on neutrophil phagocytic activity or oxidative burst responses to bacterial pathogens in non-human primates and healthy volunteers, respectively (61, 63). Interestingly, it was reported that *CXCR2* deficiency impairs neutrophil migration toward CXCR2 ligands, while their ability to migrate towards another neutrophil chemoattractant, fMLP, remains unaffected (24). These findings imply that *CXCR2*-deficient neutrophils may still be effectively recruited by alternative chemoattractant signals, such as fMLP, and possibly other chemoattractive factors that are typically generated at infection sites during bacterial challenges. This observation raises the possibility that treatments aimed at promoting neutrophil release into peripheral blood may enhance their emigration into infected tissues, thereby aiding in bacterial eradication. Supporting this hypothesis, our data indicate that treatment with a CXCR4 antagonist, which normalized the accumulation of mature neutrophils in BM, promoting their release into peripheral blood, alleviated pneumonia severity, lowered bacterial counts in the lungs, reduced mortality, and restored lung neutrophil levels. The ability of the CXCR4 antagonist to reduce pneumonia in our CXCR2 LOF mice is likely related to its ability to mobilize neutrophils and facilitate their emigration into infected tissues rather than directly influencing neutrophil effector functions. Our previous studies support this argument, demonstrating that the CXCR4 antagonist effectively mobilizes neutrophils while preserving their effector functions in a mouse model of WHIM syndrome as well as in patients with chronic neutropenia (34, 38).

To our knowledge, this is the first study to demonstrate the remarkable effects of CXCR4 antagonist treatment in reducing infection susceptibility in mice with CXCR2 LOF. Our findings in CXCR2 LOF mice are consistent with previous reports in patients with WHIM syndrome indicating that CXCR4 antagonist treatment results in clinically significant improvements in peripheral blood neutrophil levels and reductions in infection rates (64). Furthermore, we observed that the CXCR4 antagonist also enhanced neutrophil recruitment to the lungs without significantly impacting infection susceptibility in control mice. This raises questions regarding the extent of neutrophil recruitment necessary for effective bacterial clearance and whether a threshold exists below which diminished recruitment compromises lung immunity against *S. pneumoniae*. Interestingly, although the CXCR4 antagonist alone did not significantly affect bacterial load or mortality during the acute phase of infection compared to the vehicle control, we observed a trend toward improved overall survival when animals were monitored up to day 14 post-infection. This suggests that, as the infection progresses, ongoing treatment with the CXCR4 antagonist may help restore immune balance, facilitate inflammation resolution, support stem cell mobilization for lung repair, and reduce fibrosis (65–69), ultimately promoting tissue recovery and enhancing overall survival. Future studies are warranted to explore these hypotheses and clarify the mechanisms underlying the observed effects further. In conclusion, this study provides compelling evidence that CXCR4 antagonism effectively mitigates pathogenic phenotypes in a pharmacologically induced CXCR2 LOF mouse model. The observed corrections include the reversal of peripheral blood neutropenia, normalization of reduced splenic neutrophil counts, correction of mature neutrophil accumulation within BM, restoration of the M/E ratio as well as a reduction in the frequency of MK-like neutrophils in BM and the incidence of mice exhibiting neutrophil myelokathexis phenotype. Furthermore, our results demonstrate that CXCR4 antagonism reduces the severity of pneumonia in the CXCR2 LOF mice and facilitates the emigration of neutrophils into bacterially infected lung tissue. These findings highlight the potential of CXCR4 antagonist therapy as a promising strategy for improving peripheral blood neutropenia and other clinical manifestations in individuals with CXCR2 deficiencies, as well as in various subsets of SCN.

## Data Availability

The raw data supporting the conclusions of this article will be made available by the authors upon reasonable request.
