# Supplementary material for: CXCR4 antagonism corrects neutrophil abnormalities and reduces pneumonia severity in a pharmacological mouse model of CXCR2 loss-of-function-mediated neutropenia
Source: Front Immunol. 2025 Dec 10;16:1658987. doi: 10.3389/fimmu.2025.1658987 (PMC12727938; doi:10.3389/fimmu.2025.1658987)
Supplement: Supplementary file 1 [file Table1.docx]

Supplementary Material

[1. Supplementary Methods 2](#_Toc204175721)

[2. Supplementary Tables 5](#_Toc204175722)

[3. Supplementary Figure Legends 9](#_Toc204175723)

# 1. Supplementary Methods

**1.1. Ligand-binding inhibition assay**

Jurkat cells were washed once with assay buffer (Hanks’ balanced salt solution + 20 mM 4-(2-hydroxyethyl)-1-piperazineethanesulfonic acid buffer + 0.2% bovine serum albumin, pH 7.4) and then incubated for 15 minutes at room temperature (RT) with test compound diluted in assay buffer at dose-dependent concentrations. Subsequently, human C-X-C motif chemokine ligand 12–AlexaFluor647 (CXCL12-AF647; 26 ng/mL) (Almac) was added to the compound-pre-incubated cells. The cells were incubated for 30 minutes at RT. Thereafter, the cells were washed twice in assay buffer, fixed with 1% paraformaldehyde in phosphate-buffered saline, and analyzed by flow cytometry (CytoFLEX Flow Cytometer, Beckman Coulter Life Sciences). Mean fluorescence intensity of CXCL12-AF647 was determined (FCS Express™ software, De Novo Software). The percentage of inhibition was calculated according to the formula:

$$\left( 1-\frac{MFI-{MFI}_{NC}}{{MFI}_{PC}-{MFI}_{NC}} \right)\times100$$

where MFI is the mean fluorescence intensity of cells in the presence of an inhibitor, MFI_NC_ is mean fluorescence intensity of cells in the absence of the ligand, and MFI_PC_ is the mean fluorescence intensity of cells in the presence of the ligand alone.

For receptor occupancy experiments, Jurkat cells were pre-incubated with compounds at their 90% inhibition concentration for ligand-binding inhibition for 15 minutes at RT. Then, cells were washed once in assay buffer, and the compounds were let to dissociate at RT for the various predefined timepoints. Subsequently, human CXCL12-AF647 was added to the preincubated cells. After that, cells were washed twice with assay buffer and fixed with 1% paraformaldehyde. MFI of CXCL12-AF647 was determined (FCS Express™). The staining intensity was inversely proportional to the fraction of receptors occupied by the compound at the respective timepoints. Receptor occupancy half-life was calculated using the “Dissociation - One phase exponential decay” method (GraphPad Prism 10 software, GraphPad Software).

**1.2. Calcium mobilization assay**

Stable K562 clones expressing CXCR4 (1 × 10^5^ cells/well) were seeded in black 96-well plates with transparent bottom coated with poly-L-lysine (Corning) and serum-starved for 24 h. Medium was removed and cells were loaded with 100 µl of fluo-4 AM (3 µM, Invitrogen) dye solution for 45 min at 37 °C. Dye solution was prepared by diluting fluo-4 powder in DMSO + 10% Pluronic F127 (Thermo Fisher Scientific) to 3 mM and then preparing the final dilution in assay buffer (HBSS with Ca2+ and Mg2+, 20 mM HEPES, 0.375 g/l NaHCO3, 0.1% BSA + 0.77 g/L probenecid [Thermo Fisher Scientific]). Subsequently, 100 µl of assay buffer alone or assay buffer with compound dilutions was added and the plates were equilibrated in the plate reader for an additional 20 min at 37 °C. CXCL12 was injected with a simultaneous measurement of fluorescent signal (FlexStation® 3 Multi-Mode Microplate Reader; Molecular Devices). Raw traces were analyzed in SoftMax Pro 7 Software (Molecular Devices). The arbitrary units were calculated as the difference between maximal and minimal signal after treatment injection, normalized to the baseline signal before injection. Half-maximal inhibitory concentrations (IC_50_) were calculated using Prism software (GraphPad).

**1.3. Chemotaxis**

Cell migration toward CXCL12 gradient was determined using the Transwell migration assay. Neutrophils were isolated from peripheral blood samples of a healthy donor using density gradient centrifugation with the Ficoll separation method. Neutrophils were stained with 500 nM Calcein AM (Invitrogen) and pre-incubated with compound 1 for 15 minutes before transfer (5 × 10^5^ cells) to an upper well of a 5.0 µM pore size Transwell® (Corning). The lower chamber contained either CXCL12 (10 nM) in medium supplemented with 1% FBS. After 4 hours of incubation, cells that migrated to the lower chambers were collected and resuspended in Dulbecco’s phosphate-buffered saline (DPBS) containing Precision Count Beads™ (BioLegend). Migrated cells and counting beads were counted by flow cytometry. Half-maximal inhibitory concentrations (IC_50_) were calculated using Prism software (GraphPad).

**1.4. PathHunter® β-Arrestin Assay**

The effect of compound 1 on β-arrestin recruitment to the GPCR was evaluated using the PathHunter® β-Arrestin Assay (Eurofins). PathHunter cells were seeded in a total volume of 20 μL into white walled, 384-well microplates and incubated at 37°C for the appropriate time prior to testing. For antagonist determination, cells were pre-incubated with 10 µM compound 1 for 30 min followed by respective chemokine ligand challenge at the EC_80_ concentration at 37°C or room temperature for another 90 or 180 minutes. For agonist determination, 5 μL of intermediate dilution of sample stocks (5X) was added to cells and incubated at 37°C or room temperature for 90 to 180 minutes. After that, 5 μL of EC_20_ agonist in assay buffer was added to the cells and further incubated at 37°C or room temperature for another 90 or 180 minutes. Vehicle concentration was 1%. At the end of incubation, 12.5 or 15 μL (50% v/v) of PathHunter detection reagent cocktail was added to cells and further incubated for one hour at room temperature. The Chemiluminescent signal were measure using a PerkinElmer Envision TM instrument. Compound activity was analyzed using CBIS data analysis suite (ChemInnovation, CA).

For antagonist mode assays, the percentage inhibition was calculated using the following formula:

% Inhibition =100% x (1 - (mean RLU of test sample - mean RLU of vehicle control)

/ (mean RLU of EC80 control - mean RLU of vehicle control))

For agonist mode assays, the percentage activity was calculated using the following formula:

% Activity =100% x (mean RLU of test sample - mean RLU of vehicle control) / (mean

MAX control ligand - mean RLU of vehicle control)

**1.5. Pharmacokinetic studies**

Male SD rat (JH Laboratory Animal Co. LTD), weighing 244-256 g, were quarantined for 1 week before the experiment. Rat had free access to food and water. Compound 1 dissolved in 50 mM citrate buffer (pH 4.0) was given to rat via oral gavage (N = 3). The animals were anesthetized via isoflurane at the designated timepoints (before dosing and 0.25, 0.5, 1, 4, 8, and 24 hours after dosing). Approximately 150 µL of blood was taken from the animals via tail vein into K2EDTA tubes. Blood samples were put on wet ice and centrifuged to obtain plasma samples (2000 g, 5 minutes, under 4 ℃) within 15 minutes. Plasma samples were stored at approximately −70 ℃ until analysis. The plasma samples were analyzed by liquid chromatography–tandem mass spectrometry (LC-MS/MS-28) (Triple Quad™ 6500^+^ System, SCIEX, Framingham, MA, USA). An aliquot of 5 µL sample was added to 500 µL acetonitrile, which contained 50 ng/mL of an insulin secretagogue (glipizide). The mixture was vortexed for 10 minutes and centrifuged at 5800 rpm for 10 minutes. An aliquot of 0.5 µL supernatant was injected for LC-MS/MS analysis. Pharmacokinetic parameters were estimated via a noncompartmental model using Phoenix WinNonlin™ 6.4 (Certara, USA).

**1.6. Immunohistochemistry staining**

At the end of the treatment period, spleens were harvested and fixed in 4% paraformaldehyde overnight. The samples underwent a sequential dehydration process using graded ethanol concentrations and were subsequently treated with xylene before being embedded in paraffin. Tissue blocks were sectioned to a thickness of 5 µm and mounted on glass slides. Paraffin sections were subjected to antigen retrieval in a citrate buffer at 99 °C for 20 minutes. Endogenous peroxidase and non-specific antibody binding were blocked using 3% H₂O₂ and 5% normal goat serum for 10 and 15 minutes at room temperature, respectively. Afterwards, slides were incubated with a primary antibody targeting Ly6G (clone E6Z1T; Cell Signaling Technology; diluted 1:2400), a neutrophil-specific marker, for 60 minutes at room temperature. The sections were then washed in PBS and incubated with a horseradish peroxidase (HRP)-conjugated secondary antibody (TM HRP-Polymer anti-Rabbit IHC Kit KIT-5004, MaxVision) for 15 minutes at room temperature. The signal was developed using a DAB substrate (DAB kit, Maixin) for 5 minutes at room temperature, followed by counterstaining with hematoxylin for 10 seconds. Slides were scanned and quantified using the Aperio AT2 scanner. H-score was automatically calculated based on staining intensity and percentage of stained cells.

# 2. Supplementary Tables

SUPPLEMENTARY TABLE S1 Antibodies used for flow cytometry.

| Mouse flow cytometry reagent | | | |
| --- | --- | --- | --- |
| Reagent | | | Supplier |
| ACK Lysing buffer | | | Thermo Fisher |
| Live/dead | | | Thermo Fisher |
| Mouse Fc block | | | BD Biosciences |
| Fixation Solution | | | BD Biosciences |
| Staining buffer | | | eBioscience |
| Mouse flow cytometry antibody | | | |
| Antibody | Clone | Fluorescence | Supplier |
| CD45 | 30-F11 | APC | Biolegend |
| CD11b | M1/70 | BV605 | Biolegend |
| Ly6C | HK1.4 | PE-CF594 | Biolegend |
| Ly6G | 1A8 | BV421 | BD Biosciences |
| Siglec-F | E50-2440 | PE | BD Biosciences |
| CD115 (CSF-1R) | AFS98 | BV421 | Biolegend |
| CD101 | Moushi101 | PE-Cy7 | Invitrogen |
| CD45R/B220 | RA3-6B2 | PE-DAZZLE 594 | Biolegend |
| CD3 | APC-cy7 | 145-2C11 | Biolegend |
| CD4 | GK1.5 | FITC | Biolegend |
| CD8 | 53-6.7 | PerCP-Cy5.5 | BD Biosciences |
| CD49b | DX5 | PE | Biolegend |
| F4/80 | BM8 | BV510 | Biolegend |
| CD117（c-kit） | 2B8 | APC | Biolegend |
| Sca-1 | D7 | BV421 | Biolegend |
| CD16/CD32 | 93 | FITC | Biolegend |
| CD34 | SA376A4 | PE | Biolegend |

SUPPLEMENTARY TABLE S2. In vitro pharmacology profile of compound 1.

| Parameter | Compound 1 |
| --- | --- |
| Molecular weight | ~ 400 g/mol |
| CXCL12 binding inhibition (IC_50_)* | 0.7 nM |
| CXCL12-induced calcium mobilization inhibition (IC_50_)** | 3.6 nM |
| Neutrophil chemotaxis inhibition (IC_50_)*** | 7.0 nM |
| Receptor occupancy half-life* | 36 minutes |

**Jurkat cells expressing CXCR4^WT^*

*** K562 cells expressing CXCR4^WT^*

**** Neutrophil isolated from healthy volunteers*

**SUPPLEMENTARY TABLE 3. Effects of compound 1 on β-arrestin recruitment to chemokine receptors, as determined by the PathHunter® β-Arrestin Assay (Eurofins)**

| GPCR ID | Conc [µM] | Antagonist mode | | Agonist mode | |
| --- | --- | --- | --- | --- | --- |
|  |  | Assay Mode | % Inhibition | Assay Mode | % Activity |
| CXCR1 | 10 | Antagonist | 1% | Agonist | 0% |
| CXCR2 | 10 | Antagonist | 0% | Agonist | -2% |
| CXCR3 | 10 | Antagonist | -10% | Agonist | 23% |
| CXCR4 | 10 | Antagonist | 102% | Agonist | -7% |
| CXCR5 | 10 | Antagonist | -2% | Agonist | 2% |
| CXCR6 | 10 | Antagonist | 26% | Agonist | -3% |

SUPPLEMENTARY TABLE S4. Pharmacokinetic parameters following single oral administration of compound 1 and subcutaneous injection of AMD3100 in rat.

| Parameter | Compound 1 | AMD3100* | AMD3100* |
| --- | --- | --- | --- |
| Dose (mg/kg) | 10 | 1 | 12.1 |
| T_max_ (h) | 0.5 | 0.5 | 1 |
| C_max_ (ng/mL) | 83 | 1550 | 14125 |
| T_1/2_ (h) | 6.1 | 0.9 | 1.16 |
| AUC_last_ (hr×ng/mL) | 756 | 2720 | 35095 |
| V_d_ (mL/kg) | 39200 | 477 | 521 |

***** Pharmacokinetic data of AMD3100 were curated from European Medicines Agency. CHMP ASSESSMENT REPORT FOR Mozobil (2009). [https://www.ema.europa.eu/en/documents/
assessment-report/mozobil-epar-public-assessment-report_en.pdf](https://www.ema.europa.eu/en/documents/assessment-report/mozobil-epar-public-assessment-report_en.pdf) [Accessed July 2025] and Zuk et al. 2014 (1). AUC_last_, the total area under the curve from time zero to the last evaluated time point; C_max_, maximum concentration of drug in blood plasma; T_1/2_, half-life; T_max_, time after drug administration at which peak plasma concentration occurs; V_d_, volume of distribution.

1. Zuk A, Gershenovich M, Ivanova Y, MacFarland RT, Fricker SP, Ledbetter S. Cxcr(4)Antagonism as a Therapeutic Approach to Prevent Acute Kidney Injury. *Am J Physiol Renal Physiol* (2014) 307(7):F783-97. Epub 2014/08/01. doi: 10.1152/ajprenal.00685.2013.

# 3. Supplementary Figure Legends

SUPPLEMENTARY FIGURE S1

Effect of CXCR4 antagonist on RBC and platelet counts in a pharmacological mouse model of CXCR2 LOF. Absolute (**A**) RBC and (**B**) platelet counts were determined in the blood of CXCR2 and CXCR4 antagonist-treated mice 4 hours after the last dose on day 7. Data are represented as mean + SEM with 6 mice per group. Statistics were calculated using the two-way ANOVA followed by Bonferroni post hoc test. CXCR4, C-X-C chemokine receptor 4; CXCR2, C-X-C chemokine receptor 2; SEM, standard error of the mean; Veh., vehicle; CXCR4i, CXCR4 antagonist; CXCR2i, CXCR2 antagonist.

SUPPLEMENTARY FIGURE S2

Effect of CXCR4 antagonist on hematopoietic stem and progenitor and immature neutrophil counts in a pharmacological mouse model of CXCR2 LOF. (A) Gating strategy for isolation of HSC, CMP, GMP by flow cytometry. (**B**) Absolute counts of BM hematopoietic stem cells (HSC), (**C**) Common Myeloid Progenitor (CMP) and (D) Granulocyte-monocyte progenitor (GMP) and (**E**) immature neutrophils were determined 4 hours after the last dose on day 7. Data are represented as mean + SEM with 6 mice per group. Statistics were calculated using the two-way ANOVA followed by Bonferroni post hoc test. * p < 0.05, ** p < 0.01, *** p < 0.0001. CXCR4, C-X-C chemokine receptor 4; CXCR2, C-X-C chemokine receptor 2; SEM, standard error of the mean; Veh., vehicle; CXCR4i, CXCR4 antagonist; CXCR2i, CXCR2 antagonist. HSC defined as CD49b^-^B220^-^CD3^-^CD45^+^Sca-1^+^c-Kit^+^; CMP defined as CD49b^-^B220^-^CD3^-^CD45 Sca-1^-^c-Kit^+^CD34^+^CD16/32^-^; GMP defined as CD49b^-^B220^-^CD3^-^CD45 Sca-1^-^c-Kit^+^CD34^+^CD16/32^+^; Immature neutrophils defined as CD49b^-^B220^-^CD3^-^Siglec-F^-^CD115 LY6C^int^Ly6G^+^CD11b^+^CD101^-^

SUPPLEMENTARY FIGURE S3

CXCR4 antagonist normalizes splenic neutrophil count abnormalities. (**A**) Representative immunohistochemical staining of the neutrophil marker Ly6G in splenic sections from mice treated with CXCR2 and CXCR4 antagonists. Samples were collected 4 hours after the final dose on day 7. Ly6G⁺ neutrophils are identified by brown DAB staining and counterstained with blue hematoxylin. (**B**) Quantification of neutrophil content in splenic sections from the same treatment groups. Whole-slide images were acquired using the Aperio AT2 scanner, and H-scores were automatically calculated based on staining intensity and the proportion of Ly6G-positive cells. Data are represented as mean + SEM with 6 mice per group. Statistics were calculated using the two-way ANOVA followed by Bonferroni post hoc test. * p < 0.05, ** p < 0.01. CXCR4, C-X-C chemokine receptor 4; CXCR2, C-X-C chemokine receptor 2; SEM, standard error of the mean; Veh., vehicle; CXCR4i, CXCR4 antagonist; CXCR2i, CXCR2 antagonist.

SUPPLEMENTARY FIGURE S4

Effect of CXCR4 antagonist on the frequency of MK-like neutrophil in peripheral blood in a pharmacological mouse model of CXCR2 LOF. (**A**) Represent MK-like neutrophil in blood smears of control and CXCR2 LOF mice. Blood smears were collected 4 hours after the last dose on day 7 and stained with May-Grunwald Giemsa. Scale bar: 7 µm. (**B**) Frequency of MK-like neutrophil in peripheral blood of control and CXCR2 LOF mouse. Data are represented as mean + SEM with 6 mice per group. Statistics were calculated using the two-way ANOVA followed by Bonferroni post hoc test. CXCR4, C-X-C chemokine receptor 4; CXCR2, C-X-C chemokine receptor 2; SEM, standard error of the mean; Veh., vehicle; CXCR4i, CXCR4 antagonist; CXCR2i, CXCR2 antagonist.

**SUPPLEMENTARY FIGURE S1**


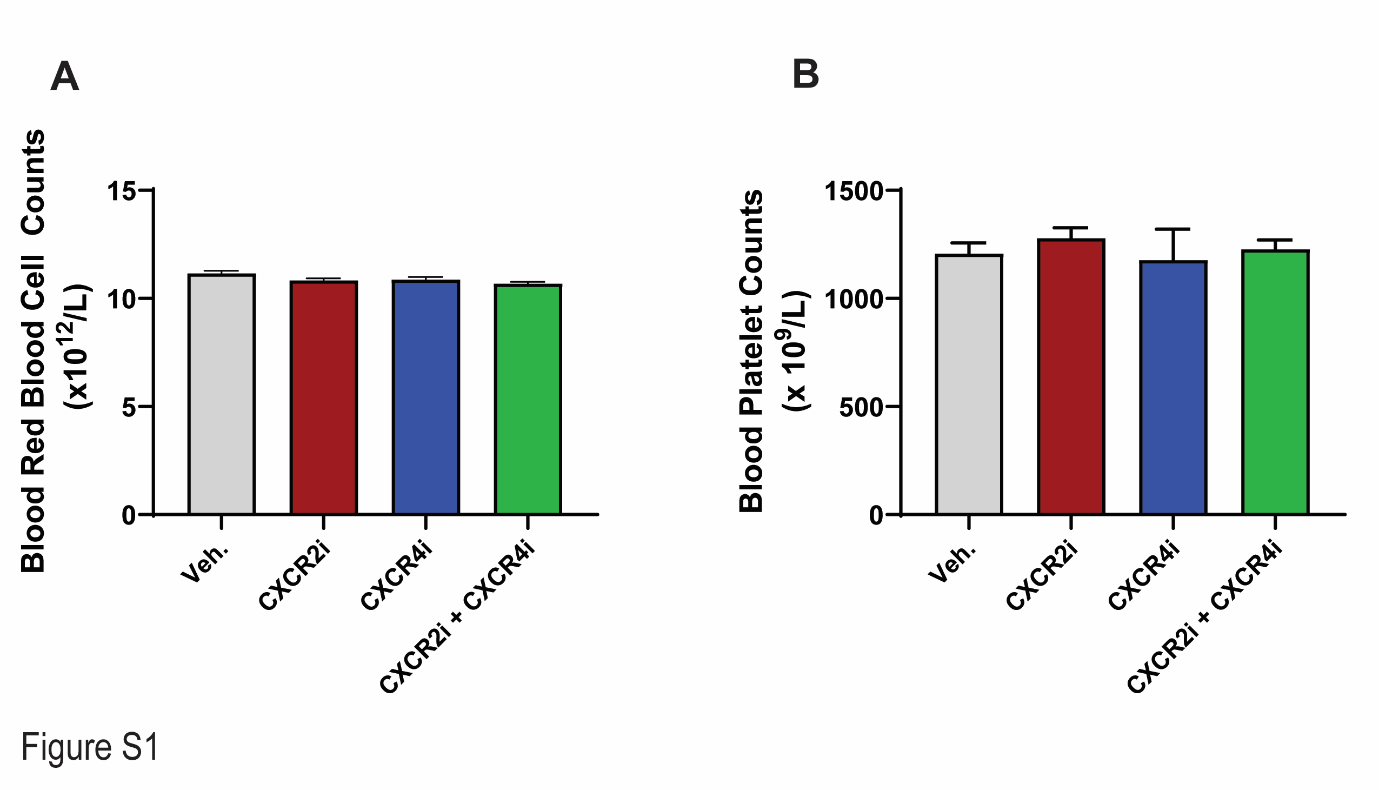


**SUPPLEMENTARY FIGURE S2**

**
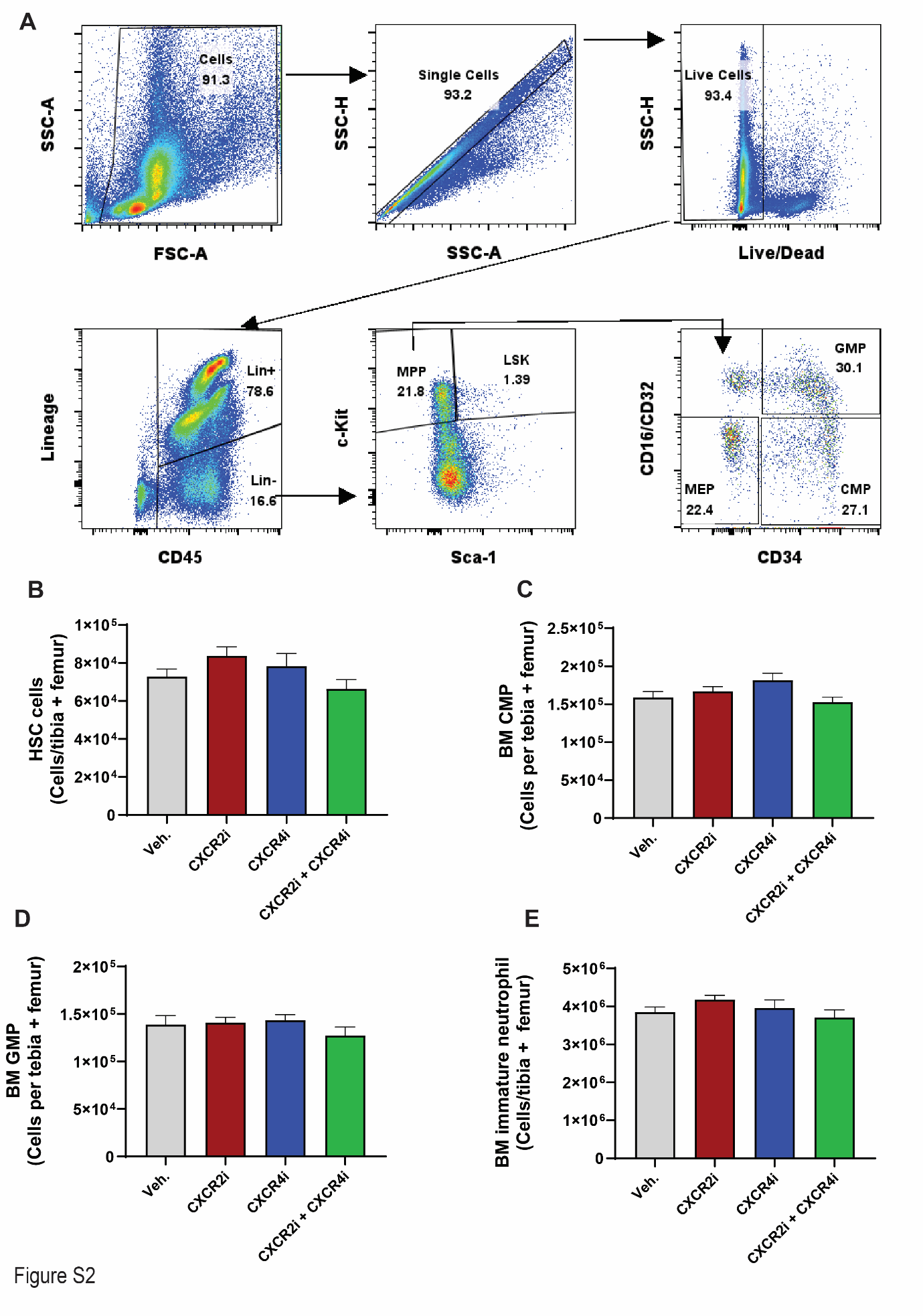
**

**SUPPLEMENTARY FIGURE S3**

**
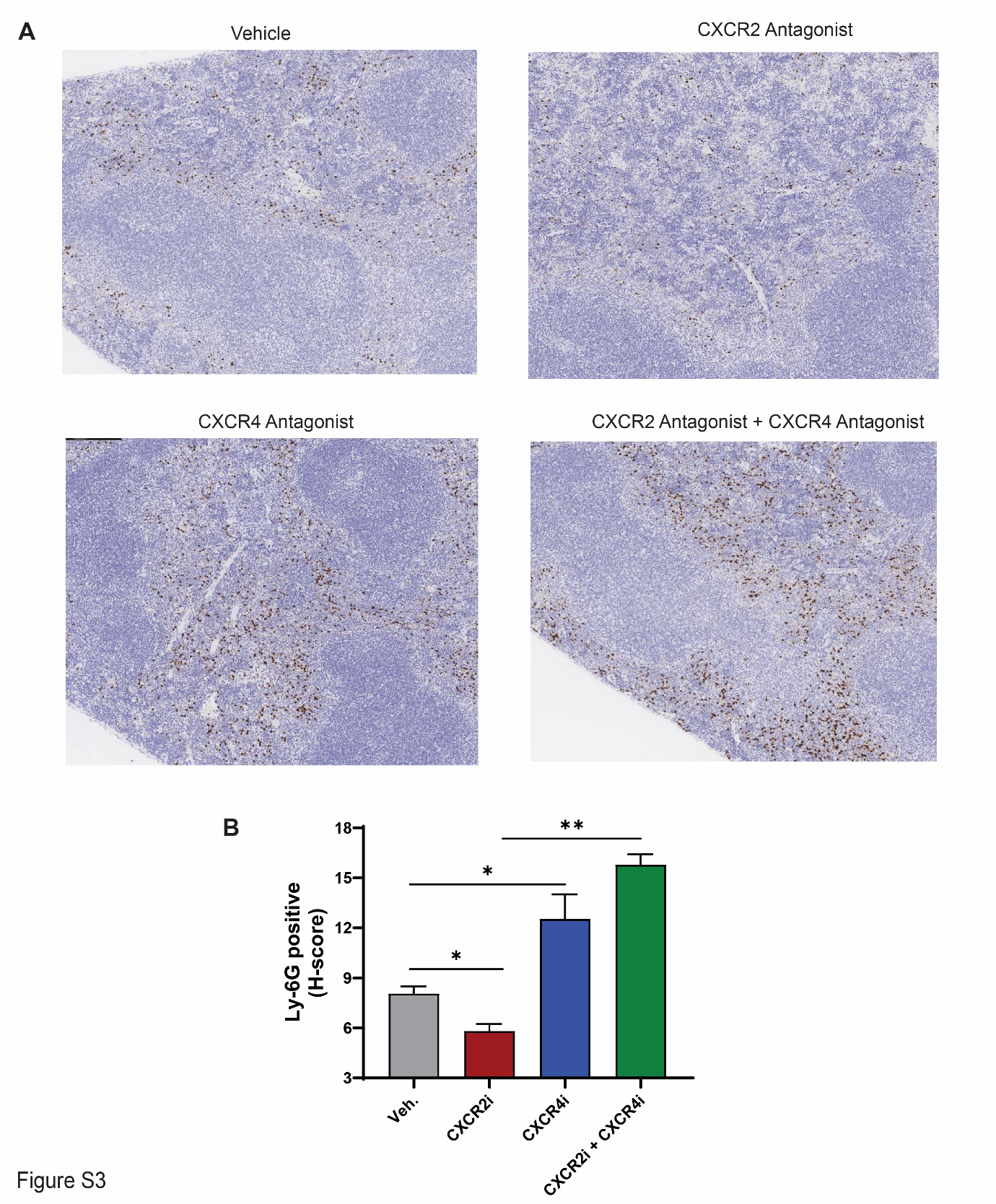
**

**SUPPLEMENTARY FIGURE S4**

**
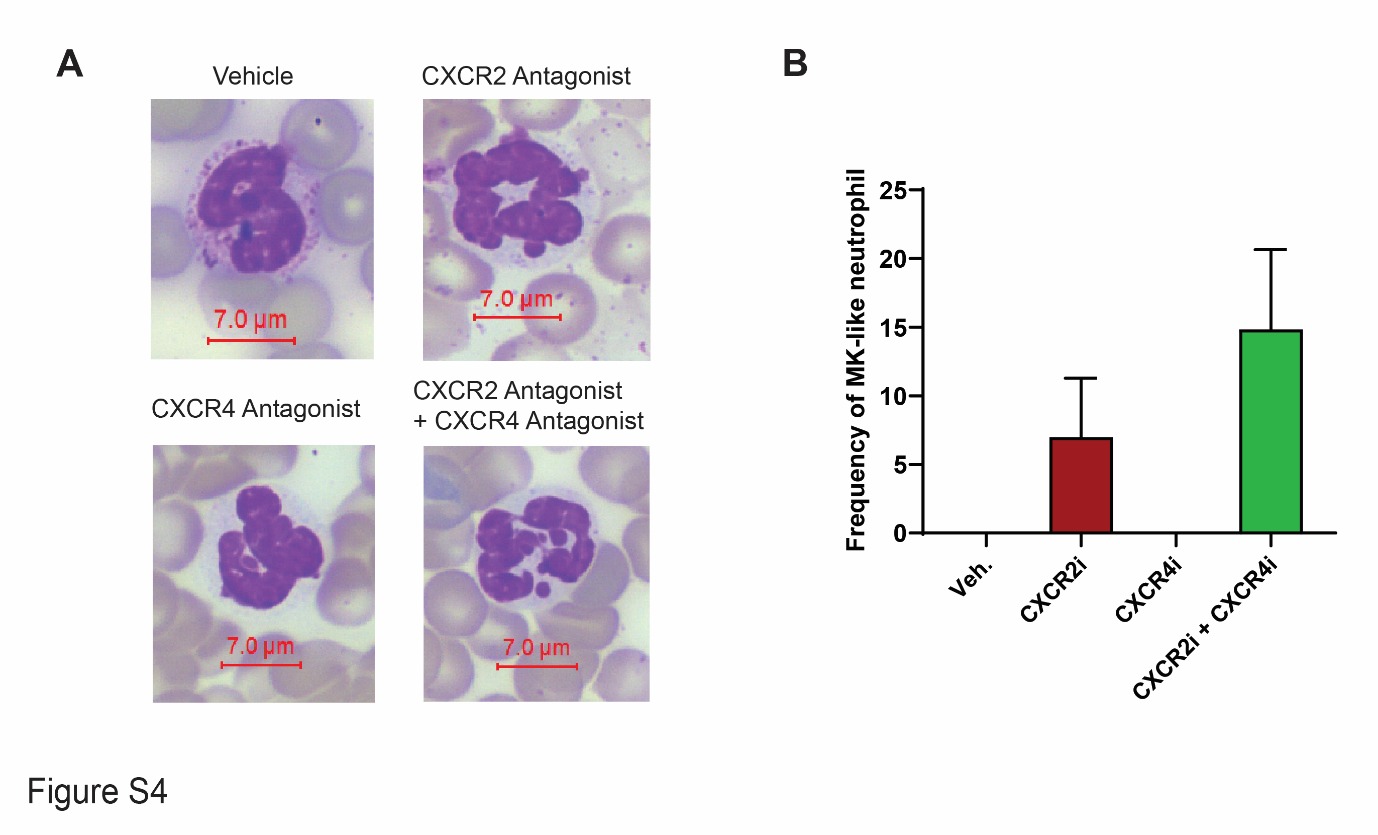
**
